# Clinical Course, Antifungal Susceptibility, and Genomic Sequencing of *Trichophyton indotineae*

**DOI:** 10.1001/jamadermatol.2024.1126

**Published:** 2024-05-15

**Authors:** Avrom S. Caplan, Gabrielle C. Todd, YanChun Zhu, Michelle Sikora, Christine C. Akoh, Jeannette Jakus, Shari R. Lipner, Kayla Babbush Graber, Karen P. Acker, Ayana E. Morales, Rebecca M. Marrero Rolón, Lars F. Westblade, Maira Fonseca, Abigail Cline, Jeremy A. W. Gold, Shawn R. Lockhart, Dallas J. Smith, Tom Chiller, William G. Greendyke, Swati R. Manjari, Nilesh K. Banavali, Sudha Chaturvedi

**Affiliations:** 1 The Ronald O. Perelman Department of Dermatology, NYU Grossman School of Medicine, New York, New York; 2 Dermatology Service, Bellevue Hospital Center, New York, New York; 3 Wadsworth Center Mycology Laboratory, New York State Department of Health, Albany; 4 SUNY Downstate Health Sciences University, Department of Dermatology, Brooklyn, New York; 5 Department of Dermatology, Weill Cornell Medicine, New York, New York; 6 Division of Infectious Diseases, Department of Pediatrics, Weill Cornell Medicine, New York, New York; 7 Division of Infectious Diseases, Department of Medicine, Weill Cornell Medicine, New York, New York; 8 Department of Pathology and Laboratory Medicine, Weill Cornell Medicine, New York, New York; 9 NYC Health + Hospitals/Lincoln Medical Center, Department of Dermatology, Bronx, New York, USA Department of Dermatology, Weill Cornell Medicine, New York; 10 Mycotic Diseases Branch, Centers for Disease Control and Prevention, Atlanta, Georgia; 11 New York City Department of Health and Mental Hygiene, New York, New York; 12 Division of Translational Medicine, Wadsworth Center, New York State Department of Health, Albany; 13 Department of Biomedical Sciences, School of Public Health, University at Albany, Albany, New York

## Abstract

**Importance:**

*Trichophyton indotineae* is an emerging dermatophyte causing outbreaks of extensive tinea infections often unresponsive to terbinafine. This species has been detected worldwide and in multiple US states, yet detailed US data on infections with *T indotineae* are sparse and could improve treatment practices and medical understanding of transmission.

**Objective:**

To correlate clinical features of *T indotineae* infections with in vitro antifungal susceptibility testing results, squalene epoxidase gene sequence variations, and isolate relatedness using whole-genome sequencing.

**Design, Setting, and Participants:**

This retrospective cohort study of patients with *T indotineae* infections in New York City spanned May 2022 to May 2023. Patients with confirmed *T indotineae* infections were recruited from 6 New York City medical centers.

**Main Outcome and Measure:**

Improvement or resolution at the last follow-up assessment.

**Results:**

Among 11 patients with *T indotineae* (6 male and 5 female patients; median [range] age, 39 [10-65] years), 2 were pregnant; 1 had lymphoma; and the remainder were immunocompetent. Nine patients reported previous travel to Bangladesh. All had widespread lesions with variable scale and inflammation, topical antifungal monotherapy failure, and diagnostic delays (range, 3-42 months). Terbinafine treatment failed in 7 patients at standard doses (250 mg daily) for prolonged duration; these patients also had isolates with amino acid substitutions at positions 393 (L393S) or 397 (F397L) in squalene epoxidase that correlated with elevated terbinafine minimum inhibitory concentrations of 0.5 μg/mL or higher. Patients who were treated with fluconazole and griseofulvin improved in 2 of 4 and 2 of 5 instances, respectively, without correlation between outcomes and antifungal minimum inhibitory concentrations. Furthermore, 5 of 7 patients treated with itraconazole cleared or had improvement at the last follow-up, and 2 of 7 were lost to follow-up or stopped treatment. Based on whole-genome sequencing analysis, US isolates formed a cluster distinct from Indian isolates.

**Conclusion and Relevance:**

The results of this case series suggest that disease severity, diagnostic delays, and lack of response to typically used doses and durations of antifungals for tinea were common in this primarily immunocompetent patient cohort with *T indotineae*, consistent with published data. Itraconazole was generally effective, and the acquisition of infection was likely in Bangladesh.

## Key Points

**Question:**

What are the clinical features, antifungal susceptibility, and genome sequencing of *Trichophyton indotineae*?

**Findings:**

In this case series of 11 patients in New York City, severe disease, ineffective standard antifungal treatments, and diagnostic delays were common. Squalene epoxidase sequence variations L393S and F397L, and terbinafine minimum inhibitory concentration values of 0.5 μg/mL or higher were associated with terbinafine therapy failure, with US isolates showing differences from known Indian isolates.

**Meaning:**

The manifestation of *T indotineae* involves extensive and recalcitrant infections, often resistant to standard terbinafine therapy, while analysis of travel history and isolate relatedness suggests a probable origin of these infections in Bangladesh.

## Teaser

This case series describes the clinical features, antifungal susceptibility, and genome sequencing of *Trichophyton indotineae* in 11 patients in New York City.

## Introduction

Dermatophytosis is a common, contagious superficial skin, hair, or nail infection caused by dermatophyte fungi,^1,2^ most commonly by members of the genus *Trichophyton*.^2,3,4^ Skin infections are often mild and resolve with topical antifungals. Systemic antifungals, such as terbinafine, a first-line oral antifungal that inhibits the enzyme squalene epoxidase (SQLE), are used to treat refractory or extensive dermatophytosis or infections involving hair follicles or nails.

In the past decade, dermatophytes failing typical doses and durations of antifungal therapy have become a global public health concern, illustrated by major outbreaks of severe, recalcitrant dermatophytosis in South Asia.^5,6,7,8,9^ These outbreaks have been driven by the emergence of a recently described species, *Trichophyton indotineae*, formerly *Trichophyton mentagrophytes* genotype VIII.^6^ *T indotineae* causes extensive, pruritic plaques, typically on the trunk, extremities, and groin,^10^ which are often minimally inflammatory, do not resolve with topical antifungals alone, and typically fail oral terbinafine treatment^7,11^ at doses and durations used for tinea infections. Clinical failure with other antifungals, including azoles and griseofulvin, are also reported.^11^ Itraconazole is currently recommended for patients who do not respond with terbinafine, but high doses and long treatment durations (>6 weeks) are required to clear *T indotineae* infections with reports of relapse occurring.^12,13^

Published data demonstrate that *T indotineae* isolates displaying terbinafine minimum inhibitory concentration (MIC) values of 0.5 μg/mL or higher are associated with treatment failure at standard doses and durations of therapy, and they harbor specific sequence variations in SQLE, including L393S and F397L.^9,14,15,16^ Daily doses of terbinafine at 500 mg per day may overcome mildly elevated terbinafine MICs,^17,18^ yet response rates vary.^19^ Isolates responsive to terbinafine therapy are correlated with SQLE alterations at position 448 (A448T).^9,16^ However, no established clinical breakpoints for antifungal medications exist for dermatophytes, and in vitro antifungal susceptibility testing (AFST) does not necessarily correspond to clinical response, creating additional treatment challenges.

In May 2023, dermatologists and public health officials reported the first 2 confirmed *T indotineae* cases in the US.^20^ Subsequently, a retrospective review of dermatophyte AFST data identified *T indotineae* in several US states, with the earliest confirmed isolate from 2017.^4^ Despite increased US spread, cases are likely underrecognized due to lack of awareness. Furthermore, *T indotineae* is a member of the *T mentagrophytes* species complex and cannot be morphologically differentiated from other members of the complex. As such, *T indotineae* is frequently misidentified as *T mentagrophytes* or *T interdigitale*.^6,20^ Molecular methods, such as DNA sequencing, are required to accurately identify *T indotineae*.^6,20^ Due to these challenges, the prevalence of *T indotineae* in the US is unknown, and published US cases lack data linking the clinical course to AFST.^4,20^ To inform approaches to diagnosis, treatment, and outcomes, we describe the largest cohort of US patients with confirmed *T indotineae* infection to date to our knowledge, correlating epidemiologic and clinical features of cases to AFST and SQLE sequence information, and modeling of terbinafine binding to SQLE. Finally, to improve our understanding of the emergence of *T indotineae* in the US, we applied WGS to determine the relatedness of isolates recovered to that of WGS sequences from Indian isolates.

## Methods

### Patient Identification and Data Collection

This case series includes all *T indotineae* isolates identified at the Wadsworth Center, New York State Department of Health, between May 2022 and May 2023. Cases were associated with 6 New York City medical centers and confirmed from testing of laboratory samples. Data were collected on patient demographics, exposure characteristics, underlying conditions, health care utilization, and treatment outcomes using a standardized case report form created by public health authorities and distributed to clinicians after *T indotineae* laboratory confirmation. Dermatophytes other than *T indotineae* were excluded. This study encompassed public health surveillance activities and is exempt according to New York State Department of Health Institutional Review Board guidelines. Informed consent was waived because the data were deidentified. Laboratory methods are described briefly herein. Additional details are available in the eMethods in Supplement 1. The Strengthening the Reporting of Observational Studies in Epidemiology (STROBE) reporting guideline was followed.

### Identification of Isolates

*T indotineae* identification was determined by DNA sequencing of the internal transcribed spacer (ITS) region. Genomic DNA from each suspected *T indotineae* isolate was extracted, followed by polymerase chain reaction amplification of the ITS region, DNA sequencing of the polymerase chain reaction product, and analysis of the sequence data using the Basic Local Alignment Search Tool. ITS sequences were submitted to GenBank with accession numbers OR483778 to OR483790 (eTable 1 and eFigure 1 in Supplement 1).

### Antifungal Susceptibility Testing

AFST was performed by a broth microdilution assay according to Reference Method M27-A3 of the Clinical and Laboratory Standards Institute guidance.^21^ MICs were determined after 96 hours of incubation at 35 °C.

### Whole-Genome Sequencing and Bioinformatic Analysis

To determine relatedness among *T indotineae* isolates, *T indotineae* genomes were sequenced, and raw sequencing reads were deposited in the Sequence Read Archive with accession numbers SRR27198731 to SRR27198741 (eTable 2 in Supplement 1). Bioinformatic analyses were performed in CLC Genomics Workbench using CLC Microbial Genomics Module software (version 23.0.4; Qiagen, Inc). Briefly, whole genome assemblies were prepared de novo for each isolate and compared with all assembled *T indotineae* genomes available in GenBank (eTable 3 in Supplement 1). Sequencing reads from *T indotineae* isolates in the US were mapped to the reference *T indotineae* isolate, TIMM20114, from India.^22^ Subsequently, single nucleotide variations (SNVs) were detected and used to construct a phylogenetic tree. From each isolate’s DNA sequence, the protein sequence of SQLE was determined and variants were identified.

### Protein Modeling and Terbinafine Docking

To characterize the molecular mechanism underpinning terbinafine resistance, an AlphaFold^23^ model for *T mentagrophytes* SQLE was used to generate a model of *T indotineae* SQLE using template-based homology modeling with Swiss-Modeler.^24^ The structure for terbinafine was obtained from its 3D model in PubChem. Terbinafine docking was performed using QuickVina2^25^ with the AutoDock Vina scoring function.^26^ To obtain an ideal starting location for terbinafine bound to SQLE, terbinafine was aligned to 2 previously solved human SQLE protein crystal structures with bound inhibitors (Protein Data Bank identifier: 6C6N and 6C6P). ChimeraX, version 1.4,^27^ was used to generate molecular figures, and Gimp (version 2.10) was used to make composite figures.

## Results

### Patient Demographics and Clinical Features

Between May 2022 to May 2023, *T indotineae* isolates from 11 unique patients from 6 different New York City medical centers were confirmed at the Wadsworth Center, New York State Department of Health (Table 1). AFST was performed against a panel of antifungal agents (Table 2). The median (range) patient age was 39 (10-65) years with 6 male and 5 female patients. Two patients (patients B and E) were pregnant. One patient had untreated lymphoma (patient H); the remainder had no immunocompromising conditions. All but 2 patients (patients B and C) reported travel in Bangladesh before acquiring a *T indotineae* infection; patient B had no travel history or known contact with an infected person, and exposure characteristic data were missing for patient C. Household transmission was considered highly likely in 3 instances and possible in another instance.

**Table 1.** Epidemiologic and Clinical Features of *Trichophyton indotineae* Infections in New York City, May 2022 to May 2023

Patient ID	Epidemiologic data				Clinical features						
					Lesion features		Therapeutics				Response
	Age, y; sex	Immunosuppressive medications or conditions	International travel^a^	Infections within household	Duration (onset to confirmation), mo	Body sites affected	Nonantifungal topicals	Topical antifungals	Oral antifungals	Oral antifungal at follow-up or resolution	Lesion status at last follow-up visit
A	50s; F	None	Bangladesh, 1 y before presentation	Unknown	6	Forearms, feet, thighs, mons pubis, buttocks, inguinal folds	Fluticasone cream, clobetasol ointment	CTZ, KCZ, ECZ, TRB	GSF, TRB, FLC	FLC	Resolved FLC, 150 mg, weekly × 4 wk
B	20s; F	Pregnant	None	None	13	Abdomen, neck, mons pubis, buttocks, inner thighs	None	TRB, KCZ	TRB, ITC	ITC	Resolved ITC, 100 mg, twice daily × 4 wk
C	<10; M	None	Unknown	Unknown	13	Abdomen, proximal legs	None	CPX, CTZ, KCZ, TRB, CPX	GSF, ITC	ITC	Unknown (lost to follow-up)
D	40s; F	None	Bangladesh, 1 mo before diagnosis	Yes, >1 family member	7	Arms, axilla, buttocks, mons pubis, inner thighs, upper legs	Hydrocortisone ointment	CTZ, TRB, KCZ	TRB, GSF	GSF	Resolved GSF, 5 mg/kg/d, × 8 wk
E	40s; F	Pregnant	Bangladesh 3 mo prior	None	6	Forearm, trunk, left axilla, left thigh	Hydrocortisone ointment	CTZ, KCZ, TRB	FLC	FLC	Resolved FLC, 200 mg, weekly × 12 wk
F	20s; M	None	Bangladesh within last 2 y	Yes, 1 family member	13	Left ear, left upper extremity trunk, neck, groin	Tacrolimus,^b^ penicillin cream^b^, triamcinolone acetonide ointment, glycerin, antiseptic solution	NFT, CTZ, ECZ, KCZ	VRC^c^, TRB, ITC	ITC	Resolved ITC, 100 mg, twice daily × 8 wk, then 200 mg 4 times daily × 4 wk
G	10s; M	None	Bangladesh within last 2 y	Yes, 1 family member	13	Face (forehead, nose, upper cutaneous lip), trunk, groin, buttocks	Triamcinolone in combination with econazole^b^	CTZ, KCZ, ECZ, TRB^c^	VRC^c^, TRB^d^, GSF, ITC	ITC	Improving while taking ITC, 100 mg, twice daily, lost to follow-up
H	60s; M	Lymphoma; untreated at diagnosis	Bangladesh 1 mo prior	Yes, 1 family member	3	Arms, thighs, buttocks, groin	None	KCZ, LCZ	ITC	ITC	Improving while taking ITC, 100 mg, twice daily
I	40s; M	None	Bangladesh 3 mo prior	None	9	Axilla, groin, buttocks	None	CTZ, KCZ	FLC, ITC	ITC	Resolved ITC, 100 mg, twice daily × 6 wk
J	30s; M	None	Bangladesh within year	Yes, >1 family member	10	Trunk, groin	Triamcinolone cream	CTZ, MCZ, KCZ	TRB, FLC, GSF	GSF^e^	Improving while taking GSF
K	20s; F	None	Bangladesh within year	>1 Family member in Bangladesh in the prior 1 y	42	Legs, upper thighs, buttocks	Hydrocortisone cream	KCZ	TRB, ITC^e^	ITC^e^	Unknown (lost to follow-up)

Abbreviations: CPX, ciclopirox; CTZ, clotrimazole; ECZ, econazole; FLC, fluconazole; GSF, griseofulvin; ID, identifier; ITC, itraconazole; KCZ, ketoconazole; LCZ, luliconazole; MCZ, miconazole; NFT, naftifine; TRB, terbinafine; VRC, voriconazole.

^a^ All patients except patient B had travel history to Bangladesh. Patient C was lost to follow-up before travel history could be obtained.

^b^ Prescribed in Bangladesh before presentation.

^c^ Prescribed in Bangladesh before presentation. Unknown dose and duration of voriconazole therapy.

^d^ Unknown if TRB was topical or oral.

^e^ ITC stopped or contraindicated due to adverse effects.

**Table 2.** Antifungal Susceptibility Testing Results for *Trichophyton indotineae* in New York City, May 2022 to May 2023

Patient (isolate) identifiers	MIC, μg/mL^a^											SQLE variant
	AMB	AND	CAS	MCF	PSC	VRC	FLC	ITC	KTC	GSF	TRB	
A (SAMN38471353)^b^	NA	NA	NA	NA	NA	NA	NA	NA	NA	NA	NA	F397L
B (SAMN38471354)	0.5	≤0.015	≤0.015	≤0.015	0.06	0.25	16	0.06	0.12	4	1	L393S
C (SAMN38471357)	0.5	≤0.015	≤0.015	≤0.015	0.06	0.25	16	0.12	0.12	4	≤0.0039	A448T
D (SAMN38471355)	0.5	≤0.015	≤0.015	≤0.015	0.12	0.5	32	0.12	0.5	4	0.5	L393S
E (SAMN38471356)	0.5	≤0.015	≤0.015	≤0.015	0.5	4	32	0.25	1	2	≤0.0039	A448T
F (SAMN38471361)	0.5	≤0.015	≤0.015	≤0.015	0.25	2	16	0.25	1	4	>128	F397L
G (SAMN38471359)	0.5	≤0.015	≤0.015	≤0.015	0.5	2	64	0.5	1	4	>128	F397L
H (SAMN38471358)	0.5	≤0.015	≤0.015	≤0.015	0.25	1	32	0.25	0.5	4	0.5	L393S
I (SAMN38471360)	1	≤0.015	≤0.015	≤0.015	0.12	1	32	0.12	0.5	4	≤0.0039	A448T
J (SAMN38471362)	0.5	≤0.015	≤0.015	≤0.015	0.06	0.25	16	0.06	0.25	2	32	F397L
K (SAMN38471363)	0.5	≤0.015	≤0.015	≤0.015	0.12	0.25	16	0.12	0.5	4	128	F397L

Abbreviations: AMB, amphotericin B; AND, anidulafungin; CAS, caspofungin; FLC, fluconazole; GSF, griseofulvin; ITC, itraconazole; KTC, ketoconazole; MCF, micafungin; MIC, minimum inhibitory concentration; NA, not applicable; PSC, posaconazole; SQLE, squalene epoxidase; TRB, terbinafine; VRC, voriconazole.

^a^ MIC values for azoles, terbinafine, and griseofulvin were defined as the lowest concentration of drug that inhibited more than 90% of growth relative to the antifungal agent-free control. MIC for echinocandins was defined as the lowest concentration of drug that led to small, rounded, compact hyphal growth relative to fuzzy and dispersed growth in the control well. The MIC value for amphotericin B was defined as the lowest concentration at which 100% of growth was inhibited.

^b^ Isolate was not saved for antifungal susceptibility testing.

**Figure 1.**
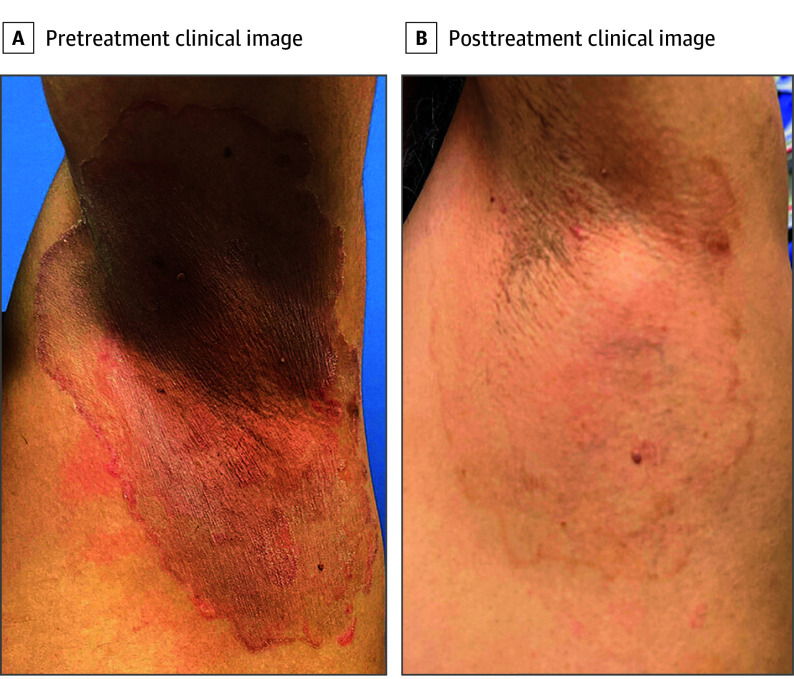
*Trichophyton indotineae* Causing Tinea Corporis The patient was treated with 6 weeks of itraconazole, 100 mg twice daily.

The median (range) time from tinea onset to diagnosis was 10 (3 [patient H] to 42 [patient K]) months. All patients had lesions affecting multiple body sites, most commonly the trunk, extremities, buttocks, and groin; 2 patients (patients F and G) had facial involvement. Nine patients reported pruritus. Six patients received medium- to high-potency topical corticosteroids before tinea diagnosis; 2 patients received the topical corticosteroid as part of combination corticosteroid-antifungal creams obtained in Bangladesh. All patients received at least 1 topical antifungal medication, none of which was effective as monotherapy. Duration of topical and oral antifungal therapy and adherence to therapy abroad before presentation were unknown. Ten of the patients received multiple topical antifungal agents. All patients demonstrated incomplete responses to typical doses and duration of oral antifungal therapy for tinea corporis/cruris (Figure 1).

Seven patients (patients A, B, D, F, G, J, and K) received a course of terbinafine without resolution of infection. Terbinafine doses were universally 250 mg daily and ranged from 14 days (patients B, J, and K) to 28 days (patients D and F) to 42 days (patient A). The dose and duration of terbinafine for 1 patient (patient G) was unknown. Seven patients’ isolates had elevated terbinafine MIC values (range, 0.5 to >128 μg/mL). AFST data and terbinafine MIC values were unavailable for patient A, as this patient’s isolate was not saved. Three patients (patients C, E, and I) who did not receive oral terbinafine treatment had isolates with terbinafine MIC values of 0.0039 μg/mL or lower.

Four patients (patients A, E, I, and J) received fluconazole treatment. Two patients (patients A and E) had a resolution while taking fluconazole; fluconazole AFST data were unavailable for patient A. The fluconazole MIC value for the isolate recovered from patient E was 32 μg/mL. Patient A received fluconazole, 150 mg, weekly for 4 weeks, and patient E received fluconazole, 200 mg, weekly for 12 weeks. Fluconazole therapy failed in 2 patients (patients I and J), with fluconazole MIC values of 32 μg/mL and 16 μg/mL, respectively. The fluconazole dose for patient I was unobtainable. Patient J received fluconazole, 200 mg, weekly for 4 weeks, without resolution.

Five patients (patients A, C, D, G, and J) were treated with griseofulvin. Patient D’s infection resolved after an 8-week course of ultramicrosize griseofulvin (375 mg/d); the griseofulvin MIC value for this patient’s isolate was 4 μg/mL. Patient J also showed improvement while taking griseofulvin after 2 months of therapy, with a MIC value of 2 μg/mL. Among the patients (patients A, C, and G) who did not respond to griseofulvin, the griseofulvin MIC values were all 4 μg/mL. Patients A and C received griseofulvin for 1 and 2 months, respectively.

Seven patients (patients B, C, F, G, H, I, and K) were treated with itraconazole, 3 of whom (patients B, F, and I), had resolution after treatment with itraconazole (dose and duration in Table 1). Two patients (patients G and H) were improving at the last known follow-up. Patient C was lost to follow-up after starting itraconazole. Patient K stopped itraconazole due to gastrointestinal adverse effects. Of the 7 patients treated with itraconazole, all had itraconazole MIC values of 0.5 μg/mL or lower. Following treatment and resolution of infection, 1 patient (patient I) developed acute urticaria and dermatographism. One patient (patient J) had contraindications to itraconazole, precluding its use. No other complications of therapy were reported. No patient received voriconazole in the US. Two patients (patients F and G) reported receiving voriconazole in Bangladesh, but the dose and duration of therapy are unknown.

### Terbinafine Antifungal Susceptibility Testing and Point Variants in Squalene Epoxidase

All 11 *T indotineae* isolates were evaluated for SQLE variants (Table 2). Five isolates harbored the point variant F397L (recovered from patients A, F, G, J, and K) and exhibited elevated terbinafine MIC values (32 to >128 μg/mL). Three isolates (recovered from patients B, D, and H) had a point variant at position 393 (L393S), corresponding to terbinafine MIC values of 0.5 to 1 μg/mL. Three isolates (recovered from patients C, E, and I) harbored a change at position 448 (A448T) and demonstrated low terbinafine MIC values, 0.0039 μg/mL or lower (Table 2).

### Phylogenetic Analyses of *T indotineae* Isolates

**Figure 2.**
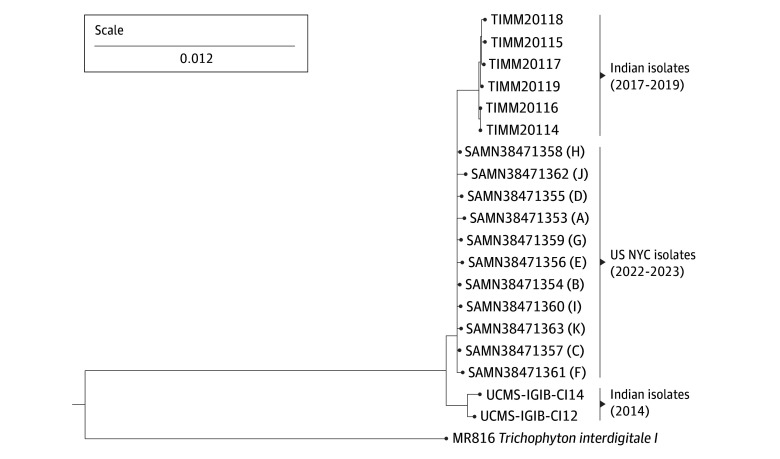
*Trichophyton indotineae* Isolates from New York City (NYC) A k-mer analysis was conducted in CLC Genomics Workbench using assembled genomes from 11 isolates recovered from patients in New York City, 8 publicly available Indian *T indotineae* genomes, and 1 publicly available *T interdigitale* genome from an isolate originating from Germany. K-mers with a prefix of ATGAC and a length of 16 on either strand were included in the analysis. The scale bar for branch length indicates the level of similarity in k-mer distribution among isolates. These isolates form a cluster distinct from *T indotineae* isolates from India.

**Figure 3.**
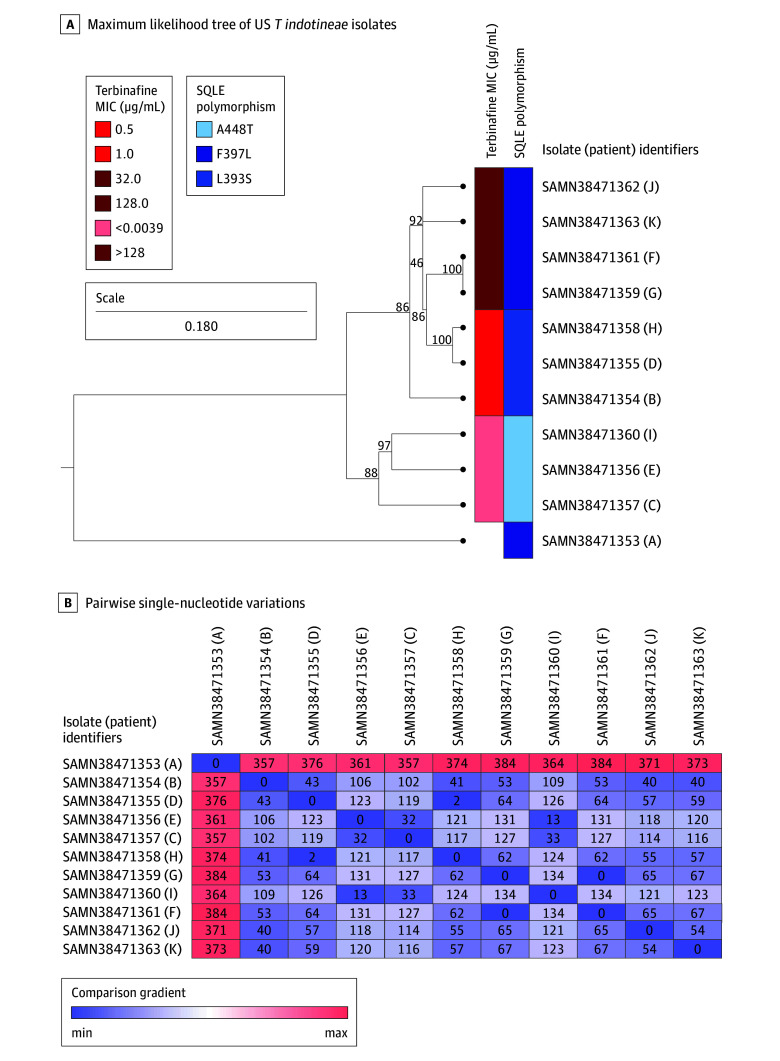
Relatedness Among New York City *Trichophyton indotineae* Isolates A, New York City *T indotineae* isolate sequencing reads were mapped to the Indian *T indotineae* reference isolate TIMM20114, and a phylogenetic tree was generated using a maximum likelihood algorithm with a Juke-Cantor nucleotide substitution model and 1000 bootstrap replicates. Bootstrap values on branches indicate the percentage likelihood that a particular branching pattern is correct. The scale bar shows the number of substitutions/changes per nucleotide. Terbinafine minimum inhibitory concentration (MIC) values and squalene epoxidase (SQLE) variants for each isolate are indicated by the red and blue bars to the right of the phylogenetic tree, respectively. B, Pairwise single nucleotide variation matrix of New York City *T indotineae* isolates. Single nucleotide variation values are color-coded along a gradient from low (blue) to high (pink). The outbreak isolates are shown from patients A to K.

A phylogenetic k-mer analysis revealed that the US isolates formed a distinct cluster from Indian isolates (Figure 2). When mapped to the most closely related Indian isolate (TIMM20114^22^), SNV analysis of the US isolates (Figure 3A) revealed that 2 patients who lived in the same household and reported previous travel to Bangladesh had identical isolates (no difference in SNVs). Two patients who lived in the same household but separately traveled to Bangladesh, had very closely related isolates (a difference of 2 SNVs). Between 32 and 373 SNVs were associated with other isolates (Figure 3B).

### Structural Changes to Terbinafine Binding Pocket in *T indotineae*

To obtain a mechanistic understanding of how SQLE variants might lead to decreased terbinafine susceptibility, an AlphaFold model of *T mentagrophytes* SQLE was used to model the terbinafine binding site in *T indotineae* SQLE using template-based homology modeling with Swiss-Modeler. The model revealed that residues L393 and F397, but not A448, form part of a hydrophobic pocket that accommodates the naphthalene moiety of terbinafine (eFigure 2 in Supplement 1).

## Discussion

To our knowledge, this case series is the largest US cohort describing *T indotineae* infections and highlights several important points. Patients experienced extensive, prolonged pruritic lesions that generally failed monotherapy with topical antifungals and showed inadequate response to typical doses and durations of oral antifungal medications, including prolonged terbinafine therapy at standard doses, consistent with findings from international reports.^5,9,15,16,28^ Diagnostic delays were common, and most patients did not have immunocompromising conditions that might predispose them to severe dermatophytosis. These findings, in addition to others (eg, recent travel to a high-prevalence region and/or initial laboratory culture identification of *T mentagrophytes* or *T interdigitale* in patients with compatible physical examination findings and history) should prompt consideration of *T indotineae*, which requires molecular-based techniques for definitive diagnosis. Because *T indotineae* identification was retrospective in most cases described in this report, treating dermatologists were often unaware of *T indotineae* diagnosis at the time of treatment, likely leading to the selection of ineffective or suboptimal antifungal treatment. Data highlight that 100-mg or 200-mg daily dosing of itraconazole for 6 to 8 weeks is the current preferred therapy, yet longer durations and higher doses may be required with reported relapse occurring.^11,12^ Terbinafine at higher than standard doses (500 mg daily) may be effective for some patients.^17,19^ Griseofulvin and fluconazole show limited efficacy.^7,13^

Consistent with published literature,^9,15,16,29^ *T indotineae* isolates recovered from patients in this study whose infections were unresponsive to terbinafine at standard doses and durations harbored variants at either position L393 (L393S; MIC values of 0.5 to 1.0 μg/mL) or position F397 (F397L; MIC values between 32 to >128 μg/mL). Three patients (patients B, D, and H) who had isolates with terbinafine MIC values of 0.5 to 1 μg/mL received terbinafine at 250 mg dosing; whether 500-mg daily terbinafine dose would have achieved cure in these patients as has been reported elsewhere is unknown.^17^ Only 3 of 11 (patients C, E, and I) patients had isolates with terbinafine MIC values in ranges reported to correlate with terbinafine susceptibility (≤0.0039 μg/mL); however, the clinical response is unknown as none was treated with terbinafine. These 3 patient isolates harbored the A448T variant, consistent with published literature.^9,30^

Itraconazole therapy did not fail in any patients in this series, yet prolonged treatment durations were required to achieve a cure. Only 1 isolate in this series had an elevated itraconazole MIC value (patient G, 0.5 μg/mL). This patient was improving while taking itraconazole but was lost to follow-up. Three patients (patients C, I, and K) isolates displayed itraconazole MIC values (0.12 μg/mL); the infection resolved successfully for 1 patient (patient I). Patient C was lost to follow-up; patient I had itraconazole-associated gastrointestinal adverse effects and stopped therapy. In 1 large study of Indian isolates, the presence of the SQLE variant A448T was associated with elevated voriconazole and itraconazole MIC values^15^; however, this finding may have been chance and requires further investigation. Compared with the data herein, of the 3 patients whose *T indotineae* isolates harbored the SQLE A448T variant (patients C, E, and I), all had voriconazole MIC values (0.25-4 μg/mL), but none were treated with voriconazole and thus clinical responsiveness could not be determined. The third patient (patient E) with SQLE A448T had an itraconazole MIC of 0.25 μg/mL but was not treated with itraconazole, so clinical responsiveness could not be determined.

Patient response to other antifungals did not correlate with antifungal MIC values. Only 2 of 4 patients treated with griseofulvin exhibited improvement, despite the low MIC values for all isolates (2-4 μg/mL). Notably, this medication is not available for use in Bangladesh, presumably the source of acquisition for most patients in this report, for comparison of clinical efficacy.^13,31^ Similarly, of 4 patients treated with fluconazole, 2 (patients A and E) were successfully cured while 2 (patients I and J) were not despite isolates from these patients having MIC values of 16 to 32 μg/mL.

The phylogenetic k-mer tree revealed that the 11 *T indotineae* isolates grouped and were distinct from *T indotineae* isolates from India. Except for the present study, no *T indotineae* WGS data are available in GenBank originating from countries outside of India at the time of acceptance of this article. Based on the recent travel history of 10 of 11 patients (Table 1), the US *T indotineae* isolates likely originated in Bangladesh suggesting that *T indotineae* may be endemic in Bangladesh. *T indotineae* easily transmits from person to person, and WGS data revealed 2 patients had identical isolates suggesting household transmission or acquisition from the same source, while 2 separate patients had very closely related isolates (only 2 SNVs difference between isolates), suggesting either transmission among household contacts or acquisition from a similar source. The other 7 isolates had 32 to 373 SNVs, which could indicate several independent introductions of *T indotineae* or variants of *T indotineae* isolates within New York City.

Based on the homology modeling of *T indotineae* SQLE, A448 resides outside of the terbinafine binding pocket, consistent with the inability of the A448T variant to change terbinafine susceptibility.^9,16^ In contrast, residues L393 and F397 form part of the hydrophobic binding site for the naphthalene moiety of terbinafine. Variants in these residues result in higher terbinafine MIC values,^9,14,15,16^ likely due to disruption of terbinafine binding. The importance of an appropriately sized hydrophobic binding pocket is supported by a study demonstrating that terbinafine is a weak, partial inhibitor of human SQLE, and modeling of terbinafine in human SQLE revealed that residues I197 and L324 did not leave sufficient room for the bulky naphthalene group of terbinafine.^32^ In *Trichophyton* species, these residues are valines with smaller hydrophobic side chains that can likely better accommodate terbinafine binding, resulting in the terbinafine susceptible phenotype. These findings^32^ along with the *T indotineae* SQLE model with L393S and F397L in the terbinafine binding site support the notion that even minor disruptions to the binding pocket’s size and hydrophobicity may lead to terbinafine resistance.

### Limitations

This study is limited by the small study size and inclusion of patients from only 1 region of the country. Furthermore, there are little published data on *T indotineae* in Bangladesh, the presumable source of infection for most of these patients, limiting the potential for comparative analyses. Additionally, this study is limited by the lack of treatment details for some patients before their presentation in New York City. Further studies are needed that encompass other geographic regions in the US, expand on clinical, epidemiologic, molecular, and mycologic information, and prospectively follow up patients with confirmed *T indotineae* infection.

## Conclusions

In this case series, we describe the largest US-based cohort to date to our knowledge of patients with *T indotineae* infection, highlighting the importance of prompt clinical recognition and the need for molecular testing to accurately identify *T indotineae*. Additionally, we review current preferred treatment approaches and correlate AFST data with *T indotineae* SQLE variants. Given the global spread of *T indotineae*, a strong international collaborative effort among clinicians, public health professionals, and clinical microbiologists is needed to characterize *T indotineae* pathophysiology and transmissibility, promote the judicious use of antifungal medications, develop standardized treatment algorithms, and monitor and mitigate the spread of emerging resistance in dermatophyte infections.

## Notes

Supplement 1. **eMethods.** Supplementary Methods **eFigure 1.** Phylogenetic Tree of *Trichophyton indotineae* Isolates Based on ITS Sequence **eFigure 2.** Model of *Trichophyton indotineae* SQLE Enzyme Bound to Terbinafine **eTable 1.** GenBank Accession Numbers of *Trichophyton* Isolates Analyzed in This Study for the Construction of ITS Phylogenetic Tree **eTable 2.** New York City *Trichophyton indotineae* WGS Reads Accession Numbers **eTable 3.** Genome Assembly Details **eTable 4.** GenBank Accesstion Number for SQLE Coding Sequences of *Trichophyton indotineae*

Supplement 2.
Data Sharing Statement
