# Supplementary material for: Clinical Course, Antifungal Susceptibility, and Genomic Sequencing of Trichophyton indotineae
Source: JAMA Dermatol. 2024 May 15;160(7):701–9. doi: 10.1001/jamadermatol.2024.1126 (PMC11097098; doi:10.1001/jamadermatol.2024.1126)
Supplement: Supplement 1. — eMethods. Supplementary Methods eFigure 1. Phylogenetic Tree of Trichophyton indotineae Isolates Based on ITS Sequence eFigure 2. Model of Trichophyton indotineae SQLE Enzyme Bound to Terbinafine eTable 1. GenBank Accession Numbers of Trichophyton Isolates Analyzed in This Study for the Construction of ITS Phylogenetic Tree eTable 2. New York City Trichophyton indotineae WGS Reads Accession Numbers eTable 3. Genome Assembly Details eTable 4. GenBank Accesstion Number for SQLE Coding Sequences of Trichophyton indotineae [file jamadermatol-e241126-s001.pdf]

Supplemental Online Content

Caplan AS, Todd GC, Zhu Y, et al. Clinical course, antifungal susceptibility, and genomic sequencing of *Trichophyton indotineae*. *JAMA Dermatol*. Published May 15, 2015. doi:10.1001/jamadermatol.2024.1126

eMethods. Supplementary Methods

eFigure 1. Phylogenetic Tree of *Trichophyton indotineae* Isolates Based on ITS Sequence

eFigure 2. Model of *Trichophyton indotineae* SQLE Enzyme Bound to Terbinafine

eTable 1. GenBank Accession Numbers of *Trichophyton* Isolates Analyzed in This Study for the Construction of ITS Phylogenetic Tree

eTable 2. New York City *Trichophyton indotineae* WGS Reads Accession Numbers

eTable 3. Genome Assembly Details

eTable 4. GenBank Accesstion Number for *SQLE* Coding Sequences of *Trichophyton indotineae*
eReferences

This supplemental material has been provided by the authors to give readers additional information about their work.

© 2024 Caplan AS et al. *JAMA Dermatology.*

eMethods

Isolates and culture media

All suspected *Trichophyton indotineae* isolates from 11 patients and other *T indotineae* and *Trichophyton* species used in this investigation are listed in Supplementary Tables 1, and 2. Media prepared in-house for culture of fungal isolates were Sabouraud dextrose agar (SDA; Becton Dickinson, Franklin Lakes, NJ, Catalog No. 210950), SDA containing chloramphenicol (50 µg/mL), and SDA containing cycloheximide (400 µg/mL). Potato dextrose agar (PDA; Becton Dickinson, Franklin Lakes, NJ, Catalog No. 213400) was prepared for culturing *Ti* for antifungal susceptibility testing (AFST).

DNA extraction and internal transcribed spacer sequencing

Genomic DNA from each *T indotineae* isolate was extracted with a QIAamp DNA Mini Kit (Qiagen; Catalog No. 51306) using a Qiacube automated extractor (Qiagen; Catalog No. 9001292). In brief, a portion of a *T indotineae* culture (~5 mm x 5 mm) gowing on solid media was removed with a sterile loop and added to an Eppendorf tube containing glass beads and lysis buffer, and tubes were incubated at 70°C for 1 h in a thermomixer shaking at 1,000 rpm. Subsequently, the fungal suspension was homogenized in a Precellys homogenizer (Bertin Technologies, France; program 5: 6500 RPM -3 × 60 seconds with 15 seconds interval) and the lysate (without beads) was transferred to a new tube and loaded into a Qiacube (Qiagen) for automated DNA extraction. Following extraction, the DNA was quantified using a Qubit (ThermoFisher Scientific, Waltham, MA; Catalog No. Q33238) according to the manufacturer's instructions, and stored at -80°C until use.

From purified genomic DNA, the internal transcribed spacer (ITS) region was amplified by PCR using the forward primer (V1827) 5′ GGAAGTAAAAGTCGTAACAAGG 3′ and the reverse primer (V50) 5′ TCCTCCGCTTATTGATATGC 3′. PCR products were sequenced using the Sanger sequencing method at the Wadsworth Center Advanced Genomic Technologies Core. The sequences were aligned in Geneious Prime (Dotmatics, Boston, MA; Version 2023.0.4), and the species was determined using the Basic Local Alignment Search Tool (BLAST) at the National Center for Biotechnology Information (NCBI). ITS sequences were submitted to NCBI for GenBank accession numbers (eTable 1and eFigure 1).

Antifungal Susceptibility Testing

Antifungal susceptibility testing (AFST) was performed using a broth microdilution assay according to Reference Method M27-A3 of the Clinical and Laboratory Standards Institute (CLSI)[1]. TREK frozen broth microdilution panels (Thermo Fisher Scientific, catalogue number CML1FNYD) were used for AFST against amphotericin B (AMB), anidulafungin (AND), caspofungin (CAS), micafungin (MCF), posaconazole (PSC), voriconazole (VRC), fluconazole (FLC), itraconazole (ITC) and ketoconazole (KTC). In-house plates of terbinafine hydrochloride (Sigma-Aldrich, St. Louis, MO; Product No PHR1298) and griseofulvin (Sigma-Aldrich St. Louis, MO; Product No. G4753) were prepared per CLSI standards. In brief, stocks of terbinafine (20 mg/mL) and griseofulvin (5 mg/mL) were prepared in dimethyl sulfoxide. The stocks were diluted in RPMI medium to achieve two-fold dilution series ranging from 0.0071813 to 256 mg/mL for terbinafine and 0.0625 to 32 mg/mL for griseofulvin. One hundred microliters of each dilution were placed in 96-well plates, and plates were stored at -80°C until use. Ten of the 11 *T indotineae* isolates (one *T indotineae* isolate was not saved) and one isolate each of *T mentagrophyte* and *T interdigitale* were grown on PDA at 30°C for six days. Growth was scraped from slants and placed in 10 mL of 0.9% saline containing 1% Tween 20. The cell suspension was allowed to stand for 10 min for large fungal particles to settle down. The supernatant was decanted, and then the cell suspension was adjusted to OD_{530} = 0.09 to 0.13. Subsequently, 400 mL of the adjusted suspension was added to 19.6 mL of RPMI medium, and 100 mL of cell suspension was placed in each well of the commercial and in-house prepared antifungal plates. Plates were incubated at 35°C for 96 hrs. For AMB, the minimum inhibitory concentration (MIC) value was defined as the lowest concentration at which there was 100% growth inhibition. For the echinocandins (AND, CAS and MCF), the MIC value was determined to be the lowest concentration of antifungal that led to the growth of small, rounded, compact hyphal forms relative to hyphal growth in the control well. The MIC values for all other antifungal agents were defined as the lowest antifungal concentration that caused a prominent decrease in growth (>90%) relative to

© 2024 Caplan AS et al. *JAMA Dermatology.*

the controls. *Candida krusei* (ATCC 6258) and *Candida parapsilosis* (ATCC 22019) were used as quality control strains.

Whole -genome sequencing

Genomic DNA (gDNA) from each isolate was prepared and quantified as described above. Approximately, 100-400 ng of gDNA was submitted to the Wadsworth Center Advanced Genomic Technologies Core for whole-genome sequencing (WGS) using an Illumina DNA Prep – (M) Tagmentation kit (Illumina, San Diego, CA; Catalog No. 20018705) for library preparation and sequenced with 2 x 148 bp or 2 x 150 bp runs on a NexSeq 500 or 1000 instrument (Illumina, San Diego, CA), respectively. *T indotineae* sequencing reads were uploaded to the NCBI Sequencing Read Archive (SRA) under the BioProject number PRJNA1046065 (eTable 2).

Bioinformatic Analyses

All bioinformatic analyses were performed in CLC Genomics Workbench (CLC-GW) with the CLC Microbial Genomics Module software (Qiagen, Inc., Redwood City, CA; Version 23.0.4). Fastq files from next generation sequencing (NGS) were imported into CLC-GW as Illumina sequencing reads. Sequencing reads were trimmed using quality scores with a quality limit of 0.05 and a maximum of two ambiguous nucleotides. Draft assemblies were prepared *de novo* and reads were mapped onto the assemblies with coverage ranging from 119-841 (eTable 3).

To determine relatedness among *T indotineae* isolates, whole genome draft assemblies for each isolate were compared to all assembled *T indotineae* genomes available on the NCBI at the time of this study. A *T interdigitale* genome was used as an outgroup (distantly related to *T indotineae*). A phylogenetic k-mer tree was generated using a neighbor-joining algorithm based on a feature frequency profile (FFP) via Jensen-Shannon divergences. Only k-mers beginning with the prefix "ATGAC" on either strand were indexed and the k-mer length was set to 16 nucleotides (Figure 2). Based on the resulting k-mer tree, the GenBank genome most closely related to New York City *T indotineae* isolates was TIMM20114, which was subsequently used as a reference strain for read mapping.

To determine single nucleotide polymorphisms (SNPs) among New York City *T indotineae* isolates, trimmed reads were mapped to the reference strain TIMM20114. Indels and structural variants were detected and used to inform local realignment and allow for basic variant detection. SNPs were filtered such that all had a minimum coverage of 10 and a minimum frequency of 35%. From these SNPs, a maximum likelihood algorithm was performed with 1,000 bootstrap replicates to construct a SNP tree and generated a matrix of SNP differences among isolates (Figure 3 A-B).

Nucleotide sequences of *T indotineae* squalene epoxidase (SQLE) were downloaded from GenBank (eTable 4), aligned, and a consensus sequence was extracted using CLC-GW. A BLAST search of a database containing assembled NYC *T indotineae* genomes was performed using the SQLE consensus sequence, SQLE sequence was extracted from each New York City *T indotineae* isolate, intron sequences (nucleotides 1286-1347) were removed, and coding regions were translated to protein, aligned, and amino acid changes were identified (Table 2).

Protein modeling and terbinafine docking

To gain a molecular understanding of the mechanism of terbinafine resistance, an Alphafold[5] model (A0A289ZCP0) for *T mentagrophytes* SQLE was used to generate a model of *Ti* SQLE using template-based homology modeling with Swiss-Modeler[6]. Terbinafine docking was performed using Quickvina2[7] with the Autodock Vina scoring function[8]. The structures for terbinafine and SQLE were prepared using the Autodock[9] prepare_ligand4.py and prepare_receptor4.py programs. The structure for terbinafine was obtained from its 3D model in Pubchem(https://pubchem.ncbi.nlm.nih.gov/compound/Terbinafine). To determine the location of terbinafine bound to SQLE, terbinafine was aligned to two previously solved human SQLE protein crystal structures with bound inhibitors (Protein Data Bank identifiers: 6C6N, 6C6P[10]) using the Matchmaker module in ChimeraX[11]. The center of mass of the inhibitor bound to human SQLE (PDB ID: 6C6N) was used as the location of the terbinafine binding site. University of California, San Fransisco (UCSF) ChimeraX version 1.4[11] was used to make the molecular figures, and GNU Image Manipulation Program (Gimp) (v 2.10, https://www.gimp.org/) was used to make composite figures (eFigure 2).

© 2024 Caplan AS et al. *JAMA Dermatology.*

eFigure 1. Phylogenetic tree of *T indotineae* isolates based on ITS sequence

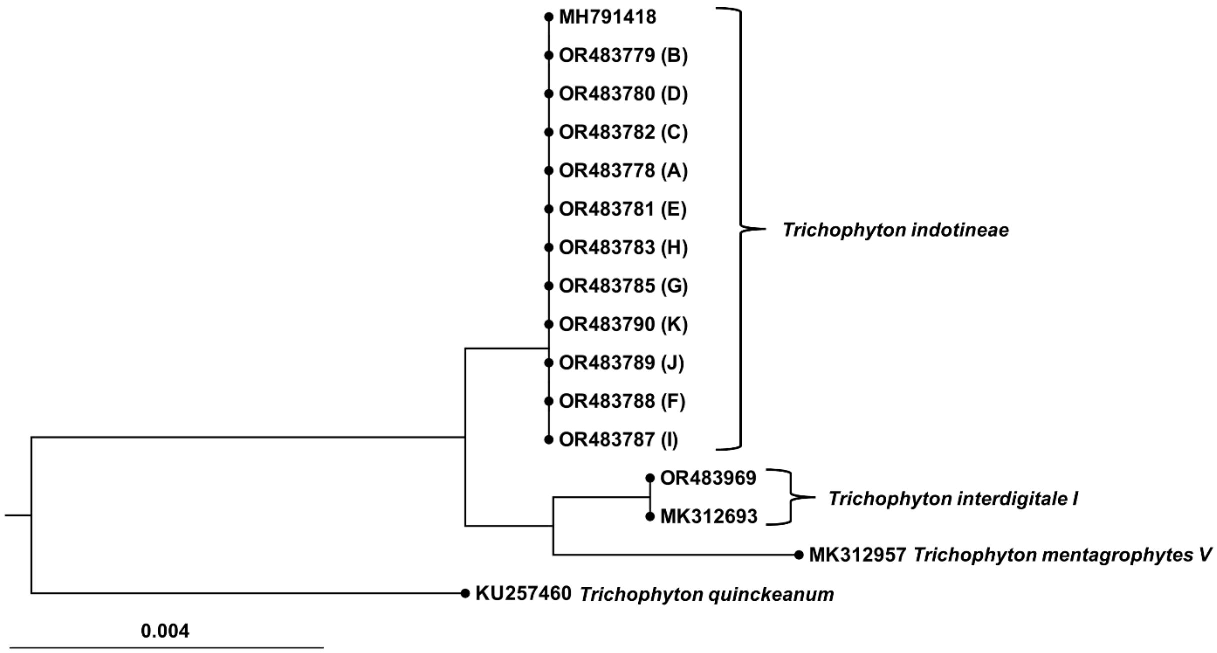

ITS sequences were aligned in CLC-GW, and a phylogenetic tree was generated using a neighbor joining algorithm with a Jukes-Cantor nucleotide substitution model and 1,000 bootstrap replicates. All New York City *T indotineae* isolates clustered together and were distinct from the closely related species *Trichophyton interdigitale* I and *Trichophyton mentagrophytes* genotype V. The *Trichophyton quinckeanum* was used as an outgroup species. The scale bar indicates the number of substitutions/changes per nucleotide.

© 2024 Caplan AS et al. *JAMA Dermatology.*

eFigure 2. Model of *T indotineae* SQLE enzyme bound to terbinafine

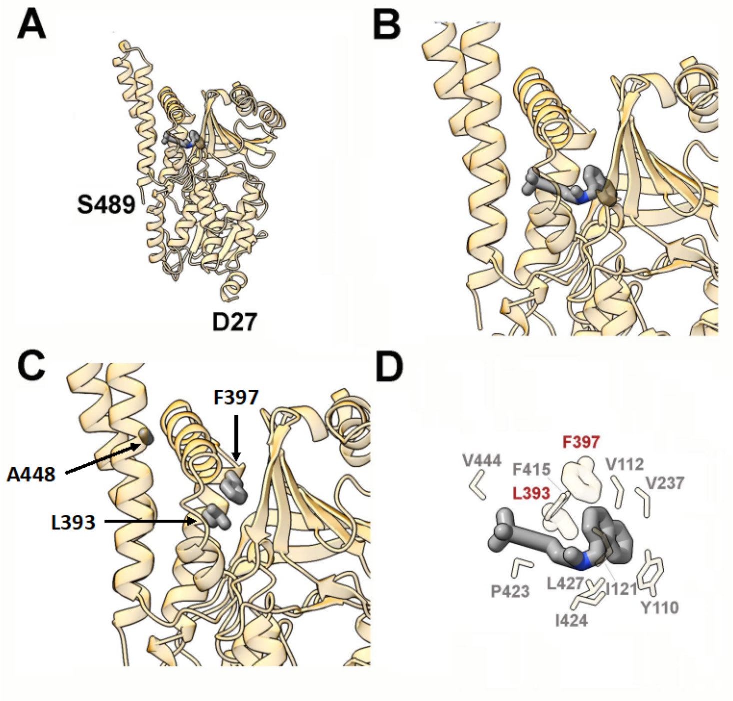

(A) Homology model of *T indotineae* SQLE rendered in transparent orange ribbons with predicted binding position of terbinafine in opaque thick stick format. **(B)** Close-up view of terbinafine bound to the predicted terbinafine binding site. **(C)** Close-up view of predicted terbinafine binding site with terbinafine removed. SQLE residues A448, L393, and F397 are rendered in opaque thick stick format. (**D**) Residues composing the terbinafine binding pocket. L393 and F397, which are implicated in binding terbinafine, are shown in thick stick format with red residue labels. Other residues predicted to interact with terbinafine are shown in thinner stick format with grey labels.

© 2024 Caplan AS et al. *JAMA Dermatology.*

eTable 1. GenBank accession numbers of *Trichophyton* isolates analyzed in this study for the construction of ITS phylogenetic tree

Sample Name	Country of Origin	GenBank Number	Genus Species	Reference
MH791418	India	MH791418	*Trichophyton indotineae*	[12]
MK312693	Iran	MK312693	*Trichophyton interdigitale* I	[13]
KU257460	Germany	KU257460	*Trichophyton quinckeanum*	[13]
A	USA	OR483778	*Trichophyton indotineae*	This study
B	USA	OR483779	*Trichophyton indotineae*	This study
C	USA	OR483780	*Trichophyton indotineae*	This study
D	USA	OR483781	*Trichophyton indotineae*	This study
E	USA	OR483782	*Trichophyton indotineae*	This study
F	USA	OR483783	*Trichophyton indotineae*	This study
G	USA	OR483785	*Trichophyton indotineae*	This study
H	USA	OR483787	*Trichophyton indotineae*	This study
I	USA	OR483788	*Trichophyton indotineae*	This study
J	USA	OR483789	*Trichophyton indotineae*	This study
K	USA	OR483790	*Trichophyton indotineae*	This study
M22570	USA	OR483969	*Trichophyton interdigitale* I	This study

© 2024 Caplan AS et al. *JAMA Dermatology.*

eTable 2. Accession numbers for New York City *T indotineae* WGS reads

Patient	Patient isolate identifier	NCBI-SRA number
A	SAMN38471353	SRR27198741
B	SAMN38471354	SRR27198740
D	SAMN38471355	SRR27198738
E	SAMN38471356	SRR27198737
C	SAMN38471357	SRR27198736
H	SAMN38471358	SRR27198735
G	SAMN38471359	SRR27198734
I	SAMN38471360	SRR27198733
F	SAMN38471361	SRR27198732
J	SAMN38471362	SRR27198731
K	SAMN38471363	SRR27198739

© 2024 Caplan AS et al. *JAMA Dermatology.*

eTable 3. Genome assembly details of New York City *T indotineae* isolates

Patient	A	B	C	D	E	
**BioSample #**	**SAMN38471353**	**SAMN38471354**	**SAMN38471357**	**SAMN38471355**	**SAMN38471356**	
**Reads**	9,187,496	12,714,268	18,686,086	13,051,138	18,859,964	
**Bases**	1,343,028,165	1,847,510,283	2,748,349,529	1,901,159,272	2,775,432,302	
**Trimmed Reads**	9,187,162	12,714,140	18,686,086	13,051,023	18,859,964	
**De Novo Assembly**						
**Mapped Reads**	9,155,846	12,684,477	18,590,576	13,018,349	18,761,747	
**Contigs**	321	188	286	209	320	
**Depth of Coverage**	144	242	358	421	227	
**Genome size Estimate**	22,136,562	22,130,599	22,127,390	22,114,581	22,121,700	
**% GC content**	48.7	48.7	48.7	48.7	48.7	

Patient	F	G	H	I		J	K
**BioSample #**	**SAMN38471361**	**SAMN38471359**	**SAMN38471358**	**SAMN38471360**		**SAMN38471362**	**SAMN38471363**
**Reads**	11,940,080	11,790,690	15,897,352	8,197,530		45,248,410	14,662,528

© 2024 Caplan AS et al. *JAMA Dermatology.*

**Bases**	1,748,027,712	1,711,772,374	2,221,019,048	1,186,100,616		5,966,455,343	2,151,286,108
**Trimmed Reads**	11,940,065	11,790,227	15,896,680	8,197,511		45,248,367	14,662,517
**De Novo Assembly**							
**Mapped Reads**	11,890,376	11,746,315	15,827,813	8,157,436		44,932,372	14,555,713
**Contigs**	352	294	299	319		492	314
**Depth of Coverage**	209	176	261	119		841	311
**Genome size Estimate**	22,127,081	22,136,995	22,133,537	22,125,541		22,147,405	22,128,159
**% GC content**	48.7	48.7	48.7	48.7		48.7	48.7

© 2024 Caplan AS et al. *JAMA Dermatology.*

eTable 4. GenBank accesstion number for SQLE coding sequences of *T indotineae*

GenBank number	Genus Species	Country of origin	Reference
ON863900	*Trichophyton indotineae*	Turkey	[14]
MN519539	*Trichophyton indotineae*	India	Ebert, A. et al. 2020, unpublished
MN519538	*Trichophyton indotineae*	India	Ebert, A. et al. 2020, unpublished
MN519537	*Trichophyton indotineae*	India	Ebert, A. et al. 2020, unpublished
MN519536	*Trichophyton indotineae*	India	Ebert, A. et al. 2020, unpublished
OL415223	*Trichophyton indotineae*	France	[15]
OL415222	*Trichophyton indotineae*	France	[15]
OL415221	*Trichophyton indotineae*	France	[15]
OL415220	*Trichophyton indotineae*	France	[15]
OL415219	*Trichophyton indotineae*	France	[15]
OL415218	*Trichophyton indotineae*	France	[15]
OM373651	*Trichophyton indotineae*	Iran	Salehi, Z. et al. 2022, unpublished
OM373650	*Trichophyton indotineae*	Iran	Salehi, Z. et al. 2022, unpublished
OM373649	*Trichophyton indotineae*	Iran	Salehi, Z. et al. 2022, unpublished
OM373648	*Trichophyton indotineae*	Iran	Salehi, Z. et al. 2022, unpublished
OM313311	*Trichophyton indotineae*	The Netherlands	[16]
OM313310	*Trichophyton indotineae*	The Netherlands	[16]
OM313309	*Trichophyton indotineae*	The Netherlands	[16]
OM313308	*Trichophyton indotineae*	The Netherlands	[16]

© 2024 Caplan AS et al. *JAMA Dermatology.*

eReferences

1. CLSI. *Reference Method for Broth Dilution Antifungal Susceptibility Testing of Yeasts*. 4th ed ed. vol CLSI standard M27. Clinical and Laboratory Standards Institute; 2017.
2. Ebert A, Monod M, Salamin K, et al. Alarming India-wide phenomenon of antifungal resistance in dermatophytes: A multicentre study. *Mycoses*. Jul 2020;63(7):717-728. doi:10.1111/myc.13091
3. Nenoff P, Verma SB, Ebert A, et al. Spread of Terbinafine-Resistant Trichophyton mentagrophytes Type VIII (India) in Germany-"The Tip of the Iceberg?". *J Fungi (Basel)*. Oct 5 2020;6(4)doi:10.3390/jof6040207
4. Shaw D, Singh S, Dogra S, et al. MIC and Upper Limit of Wild-Type Distribution for 13 Antifungal Agents against a Trichophyton mentagrophytes-Trichophyton interdigitale Complex of Indian Origin. *Antimicrob Agents Chemother*. Mar 24 2020;64(4)doi:10.1128/AAC.01964-19
5. Jumper J, Evans R, Pritzel A, et al. Highly accurate protein structure prediction with AlphaFold. *Nature*. Aug 2021;596(7873):583-589. doi:10.1038/s41586-021-03819-2
6. Schwede T, Kopp J, Guex N, Peitsch MC. SWISS-MODEL: An automated protein homology-modeling server. *Nucleic Acids Res*. Jul 1 2003;31(13):3381-5. doi:10.1093/nar/gkg520
7. Alhossary A, Handoko SD, Mu Y, Kwoh CK. Fast, accurate, and reliable molecular docking with QuickVina 2. *Bioinformatics*. Jul 1 2015;31(13):2214-6. doi:10.1093/bioinformatics/btv082
8. Trott O, Olson AJ. AutoDock Vina: improving the speed and accuracy of docking with a new scoring function, efficient optimization, and multithreading. *J Comput Chem*. Jan 30 2010;31(2):455-61. doi:10.1002/jcc.21334
9. Forli S, Huey R, Pique ME, Sanner MF, Goodsell DS, Olson AJ. Computational protein-ligand docking and virtual drug screening with the AutoDock suite. *Nat Protoc*. May 2016;11(5):905-19. doi:10.1038/nprot.2016.051
10. Padyana AK, Gross S, Jin L, et al. Structure and inhibition mechanism of the catalytic domain of human squalene epoxidase. *Nat Commun*. Jan 9 2019;10(1):97. doi:10.1038/s41467-018-07928-x
11. Pettersen EF, Goddard TD, Huang CC, et al. UCSF ChimeraX: Structure visualization for researchers, educators, and developers. *Protein Sci*. Jan 2021;30(1):70-82. doi:10.1002/pro.3943
12. Nenoff P, Verma SB, Vasani R, et al. The current Indian epidemic of superficial dermatophytosis due to Trichophyton mentagrophytes-A molecular study. *Mycoses*. Apr 2019;62(4):336-356. doi:10.1111/myc.12878
13. Uhrlass S, Verma SB, Graser Y, et al. Trichophyton indotineae-An Emerging Pathogen Causing Recalcitrant Dermatophytoses in India and Worldwide-A Multidimensional Perspective. *J Fungi (Basel)*. Jul 21 2022;8(7)doi:10.3390/jof8070757
14. Durdu M, Kandemir H, Karakoyun AS, Ilkit M, Tang C, de Hoog S. First Terbinafine-Resistant Trichophyton indotineae Isolates with Phe(397)Leu and/or Thr(414)His Mutations in Turkey. *Mycopathologia*. Jan 19 2023;188(1):2. doi:10.1007/s11046-023-00708-2
15. Moreno-Sabater A, Normand AC, Bidaud AL, et al. Terbinafine Resistance in Dermatophytes: A French Multicenter Prospective Study. *J Fungi (Basel)*. Feb 23 2022;8(3)doi:10.3390/jof8030220
16. Astvad KMT, Hare RK, Jorgensen KM, Saunte DML, Thomsen PK, Arendrup MC. Increasing Terbinafine Resistance in Danish Trichophyton Isolates 2019-2020. *J Fungi (Basel)*. Jan 31 2022;8(2)doi:10.3390/jof8020150

© 2024 Caplan AS et al. *JAMA Dermatology.*
