# Supplementary material for: Clinical Course, Antifungal Susceptibility, and Genomic Sequencing of Trichophyton indotineae
Source: JAMA Dermatol. 2024 May 15;160(7):701–9. doi: 10.1001/jamadermatol.2024.1126 (PMC11097098; doi:10.1001/jamadermatol.2024.1126)
Supplement: Supplement 2. — Data Sharing Statement [file jamadermatol-e241126-s002.pdf]

Data Sharing Statement

Caplan. Clinical Course, Antifungal Susceptibility, and Genomic Sequencing of Trichophyton indotineae. *JAMA Dermatol*. Published May 15, 2024. doi:10.1001/jamadermatol.2024.1126

Data
Data available: No
